# Serum levels of urokinase-type plasminogen activator in healthy dogs and oncologic canine patients

**DOI:** 10.14202/vetworld.2017.918-923

**Published:** 2017-08-14

**Authors:** Sofia C. Ramos, Augusto J. de Matos, João Niza Ribeiro, Liliana R. Leite-Martins, Rui R. F. Ferreira, Inês Viegas, Andreia A. Santos

**Affiliations:** 1 Department of Veterinary Clinics, Faculty of Veterinary Medicine of the University Lusófona of Humanities and Technologies, Campo Grande 376, Lisbon, Portugal; 2 Department of Veterinary Clinics of the Biomedical Sciences Institute of Abel Salazar (ICBAS), University of Porto, R. Jorge Viterbo Ferreira no. 228, Porto, Portugal; 3 Animal Science and Study Centre/Food and Agrarian Sciences and Technologies Institute (CECA/ICETA), P. Gomes Teixeira, Portugal; 4 Department of Population Studies, Biomedical Sciences Institute of Abel Salazar (ICBAS), University of Porto, R. Jorge Viterbo Ferreira no. 228, Porto, Portugal; 5 Institute of Public Health, University of Porto, R. das Taipas 135, Porto, Portugal; 6 Animal Blood Bank (BSA), R. Jorge Viterbo Ferreira no. 228, Porto, Portugal

**Keywords:** biomarker, dogs, neoplasms, serum, urokinase plasminogen activator

## Abstract

**Aim:**

Urokinase plasminogen activator (uPA) has been scarcely studied in veterinary oncology. The aim of this study was to determine the uPA serum concentrations in healthy and oncologic canine patients and to investigate its potential value as a tumor biomarker.

**Materials and Methods:**

Serum uPA concentrations of healthy and oncologic canine patients were measured by enzyme-linked immunosorbent assay. Their relationships with the dogs’ health status and tumor characteristics were analyzed through ANOVA and independent t-test.

**Results:**

There were no significant differences between mean serum values (±standard deviation) of healthy dogs (0.19±0.13 ng/ml) and oncologic canine patients (0.22±0.33 ng/ml), or between dogs with benign or malignant tumors, and with or without metastases, although the latter tended to show higher uPA serum levels.

**Conclusion:**

This is the first study describing the uPA serum levels in dogs. Although its results do not support uPA as a tumor biomarker, higher uPA levels in dogs with metastatic neoplasms may reflect the role of the enzyme in tumor progression.

## Introduction

An early detection of a neoplasm enhances the probability of therapeutic success, highlighting the importance of biomarkers identification [1]. Multiple cancer markers have been identified in human medicine and used in clinical practice, such as prostate-specific antigen in prostatic cancer [2] and steroid hormone receptors in breast cancer [3]. Members of the urokinase plasminogen activator system (uPAS) are also examples of potential cancer biomarkers related with prognosis that has been associated with several human neoplasms [4]. However, cancer biomarkers are scarcely described in veterinary medicine.

The uPAS has been involved in a variety of physiological events such as wound healing, mammary gland development/involution, and tissue regeneration [5,6]. Through events like proteolysis of the basal membrane and extracellular matrix (ECM), initiation of epithelial–mesenchymal transition and signal transduction pathways, the uPAS is also associated to pathological events, such as tumor angiogenesis, invasion, and metastasis [7-9]. The uPAS includes the urokinase plasminogen activator (uPA), its cell membrane receptor (uPAR), the tissue plasminogen activator and its two main inhibitors, the plasminogen activator inhibitor-1 (PAI-1) and -2 (PAI-2) [7,8].

When bound to the receptor, uPA catalyzes the conversion of inactive plasminogen into active plasmin [7], which can promote fibrinolytic processes [10] and proteolytic activities involved in the ECM degradation [11]. Plasmin can also participate in other regulatory mechanisms of cell behavior, such as secretion of cytokines and activation of pro-matrix metalloproteinases, promoting cellular migration and local invasion [12]. The active form of PAI-1 may inhibit uPA [6] and internalize the complex uPA/uPAR/PAI-1 for degradation [13], recycling uPAR that is then redistributed back to the cell surface, promoting its functions [14].

The secretion, function, and expression patterns of the uPAS members have been studied in a broad range of human malignancies [6], and they are examples of potential cancer biomarkers with prognostic and predictive values, already associated with several human neoplasms, such as mammary, pulmonary, and pancreatic cancer [4]. More specifically, and as an example, elevated levels of uPA catalytic activity in patients with breast cancer were associated with a shortened disease-free interval, comparing to those with lower levels of uPA activity [15-17]. Also in breast cancer, patients without lymph node metastasis and with elevated uPA and PAI-1 levels benefited from adjuvant treatment, while similar patients but with lower levels did not [17,18].

Several studies indicate that the uPAS could be a promising therapeutic oncologic target [4,8], and some of the proposed therapeutic models included the suppression of the uPAS components [19,20], the inhibition of their proteolytic activity [21,22], the interference between uPAR connection with the respective ligands [23,24] or with integrins [25], and also toxins directed to uPAS components [26,27]. However, understanding the interactions and functions of the uPA/uPAR complex are a challenging task, because the inhibition of its components seems to originate a functional compensation by other proteases, such as metalloproteinases [28].

uPA has been studied in human medicine in several sample types, including serum levels in patients with head and neck squamous cell carcinoma [29], breast cancer [30], ovarian cancer [31] and prostatic cancer [32], and their results suggest that uPA plays important roles in cancer evolution, particularly in processes such as angiogenesis and metastasis and might also be of prognostic and predictive value.

However, the uPAS is scarcely mentioned in veterinary medicine, and serum levels of uPA have never been determined in animals. uPA has been studied by immunohistochemical methods in some canine neoplasms, such as mammary tumors [33-35] and hemangiosarcomas [36,37]. Santos *et al*. [33,34] showed that both cancer cells and adjacent stroma of malignant mammary tumors significantly overexpressed uPA. In addition, the high expression of uPA by tumor stroma was significantly associated with several poor prognosis factors, such as higher histological grade, higher Ki-67 indices, higher metastatic rate, and also with shorter overall and disease-free survival. Golshahi *et al*. [35], studying the same type of neoplasm, obtained the same results by showing that an elevated uPA stromal expression was associated with poor prognosis. Anwar *et al*. [37] demonstrated that uPA and uPAR were significantly associated with cancer cells proliferation in canine hemangiosarcomas. uPAR was also studied in canine prostatic proliferative disorders, which exhibited higher expressions comparing to normal prostatic tissues [38]. Overexpression of stromal uPAS members was also found in a feline giant cell tumor of bone [39]. Taken together, these results suggest that uPA contributes for tumor aggressiveness in canine patients and constitutes a relevant prognostic factor, supporting its potential value as a tumor biomarker and as a therapeutic target, as already demonstrated in human medicine.

The determination of cancer markers in serum may be more useful than immunohistochemistry methods for the clinical setting, by being a faster and less invasive approach that may allow an early identification of patients with cancer. Hence, this study was undertaken to address the value of uPA as a serum biomarker, to distinguish cancer patients from healthy dogs. The relationships between uPA levels and clinicopathological characteristics with prognostic relevance, such as neoplasm size, histologic grade and lymph nodes invasion/distant metastasis, were also studied. In the face of the scarcity of animal serum cancer biomarkers, this study aimed to shed an initial light on the potential of uPA as a candidate for such role.

## Materials and Methods

### Ethical approval

The study protocol was approved by the Ethics Committee of the Faculty of Veterinary Medicine, University Lusófona of Humanities and Technologies (FMV-ULHT) (N56/2014).

### Samples

Blood samples were collected from both healthy dogs and canine patients diagnosed with benign and malignant neoplasms at the hospitals of two veterinary schools, FMV-ULHT, and Veterinary Hospital of Biomedical Sciences Institute of Abel Salazar of University of Porto (UPVet-ICBAS), after owner’s informed consent. Some blood samples of control dogs were also collected at the Animal Blood Bank (BSA), Porto. From all dogs information such as age, weight and gender, past medical history and, when applicable, the tumor characteristics, such as type, location and size, was collected and registered. Dogs were grouped according to the canine clinical nutrition encyclopedia [40] in small (1-9.9 kg), medium (10-29.9 kg), and large breed dogs (≥30 kg). Cutaneous neoplasms were measured and grouped according to their largest dimension in three categories: <3 cm, 3-5 cm, and >5 cm.

All animals were older than 4 years old. The healthy Group A consisted of 21 dogs (12 females and 9 males) without previous neoplastic or inflammatory diseases, no clinical evidence of disease, and normal complete blood count and serum biochemistry. The tumor Group B included 20 dogs (11 females and 9 males) with cytologically and/or histologically confirmed neoplasms. Animals with concomitant inflammatory disease or that had been submitted to any therapeutic intervention were excluded from the study. The clinical staging was assessed in all Group B dogs, based on a complete physical examination, three thoracic radiographic views, a complete abdominal ultrasound evaluation, and cytology and/or biopsy of organs suspected of metastatic involvement.

Cytological and histological diagnoses were performed by three laboratories: Laboratory of Clinical Analysis and Histopathology of ULHT, Veterinary Pathology Laboratory of the Biomedical Sciences Institute of Abel Salazar (ICBAS), University of Porto (UP), and Cytology Diagnostic Services of ICBAS-UP.

### uPA measurement in serum

Blood samples (5 ml) were collected and introduced into a serum separator tube, allowed to clot for 8 h at 2-8°C, and centrifuged at 3000 rpm for 15 min. Serum was removed and divided into four aliquots (0.5 ml each), stored frozen at −80°C until processing. All samples with visible moderate or intense hemolysis were excluded from the study.

The supernatant hemoglobin values were measured in all samples with mild hemolysis, to investigate if its presence could influence uPA levels. For that purpose, 0.02 ml of serum was pipetted to cuvettes, subsequently introduced in a quantitative measuring device (Low Hemoglobin Analyzer Meter – Scott European Corporation), and values were recorded for further analysis.

The serum levels of uPA were measured using a canine-specific uPA sandwich enzyme-linked immunosorbent assay. The assays and data calculations were performed according to the manufacturer’s instructions (MyBiosource – MBS020636). All samples were run in duplicate and optical densities (OD) were read spectrophotometrically at 450 nm in a microplate reader. The average OD of the Kit standard solutions was calculated and standard curves were established, using CurveExpert software (MyBiosource), from which the uPA serum concentrations were extrapolated.

### Statistical analysis

All data were analyzed with IBM SPSS version 23. uPA serum concentrations of controls and cases were presented as means±standard deviation (SD). For the comparative analysis of uPA values between controls/neoplasms, benign/malignant, metastatic/nonmetastatic and variables related to the animal intrinsic characteristics, cutaneous neoplasm size and the presence of hemolysis, Student’s t-test for independent samples and ANOVA were used. The significance level was established at p<0.05.

## Results

**Figure-1:**
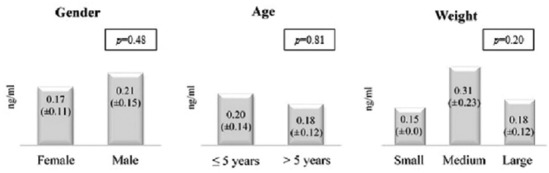
Mean urokinase plasminogen activator serum values (ng/ml) (±standard deviation) according to the intrinsic characteristics of healthy dogs.

The mean serum uPA concentration of healthy dogs (±SD) was 0.19 ng/ml (±0.13). This group was composed by 85.7% large breed, 9.5% medium breed, and 4.8% small breed dogs. The concentrations were not significantly influenced by intrinsic characteristics such as gender, age, and weight (Figure-1).

The group of oncologic patients included 5 dogs affected by benign and 15 by malignant tumors, including 7 epithelial, 5 mesenchymal, 4 hematopoietic/lymphoreticular, 1 gonadal and 3 mixed neoplasms. Their mean serum uPA concentration (±SD) was 0.22 ng/ml (±0.33). The uPA concentrations were not significantly different between healthy and diseased patients or between dogs affected by benign or malignant neoplasms (Table 1).

**Table-1:** uPA serum concentrations in healthy dogs and in oncologic canine patients.

Variable	Mean uPA serum values±SD (ng/ml)	CI (95%)		p value	Animals
	
		Minimum	Maximum		n (%)
Healthy dogs	0.19 (0.13)	0.13	0.25	0.67	21 (51)
Oncologic patients	0.22 (0.33)	0.07	0.38		20 (49)
Benign	0.17 (0.12)	0.02	0.32	0.70	5 (25)
Malignant	0.24 (0.38)	0.03	0.45		15 (75)
Metastatic	0.37 (0.54)	−0.13^a^	0.87	0.23	7 (47)
Nonmetastatic	0.13 (0.06)	0.08	0.18		8 (53)
Cutaneous neoplasms					
<3 cm	0.11 (0.03)	0.03	0.19	0.16	3 (30)
3-5 cm	0.23 (0.11)	0.04	0.41		4 (40)
>5 cm	0.11 (0.68)	−0.06^a^	0.27		3 (30)
Healthy dogs					
Nonhemolyzed	0.16 (0.06)	0.12	0.20	0.23	12 (57)
Hemolyzed	0.23 (0.18)	0.09	0.37		9 (43)
Oncologic patients					
Nonhemolyzed	0.26 (0.43)	−0.01^a^	0.53	0.52	12 (60)
Hemolyzed	0.16 (0.08)	0.09	0.23		8 (40)

a SPSS calculated value. SD=Standard deviation, CI=Confidence interval, uPA=Urokinase plasminogen activator

In the malignant group, 3 cases had lymph node metastases, 1 had distant metastases and 3 had both. Due to the small number of cases, dogs with lymph node and/or distant metastases were grouped together for statistical analysis. There were no statistical differences between dogs with or without metastases (Table 1), although the uPA values from nonmetastatic cases were 65% lower comparing to the metastatic ones.

When dogs with cutaneous neoplasms (n=10) were considered, there was no relationship between their uPA concentration and tumor size (Table 1). Finally, mild hemolysis did not significantly influence serum uPA values in either group (Table 1).

## Discussion

Considering the several evidences of the uPA role in human cancer development and progression, the main goal of this study was to determine the serum concentrations of uPA in healthy dogs and to compare them with those of cancer patients. To the best of our knowledge, this is the first study addressing this goal in canine cancer patients.

The mean uPA value of healthy dogs (0.19±0.13 ng/ml) was lower than the concentrations described in humans (0.56±0.16 to 1.1±0.38 ng/ml) [41-44]. uPA values may be species related, and perhaps uPA serum values in healthy canines are lower than in healthy humans. However, this hypothesis needs to be validated with a larger study. Nevertheless, similarly to what reported in human studies, canine uPA values seem to be independent of individual factors such as gender, age and weight [44,45].

Healthy dogs had similar uPA concentrations to oncologic canine patients, suggesting that it is unsuitable as a serum biomarker of neoplastic disease. These results are concordant with some studies of human cancer, including head and neck squamous cell carcinoma [29] and pulmonary neoplasms [41]. However, a different conclusion emerged from studies in pancreatic/biliar neoplasms [42,43], ovarian [31], and prostate cancer [32], where significant differences in uPA serum values between healthy and oncologic patients were found. Such variances suggest that circulating uPA levels may be related to the neoplasms tissue of origin or tumor histological type. Supporting this hypothesis, other studies detected a lower uPA expression in certain human tumor types, such as basal cells carcinomas [46].

In addition, the uPA values of dogs with benign neoplasms were not significantly different from those of dogs with malignant neoplasms, although it is important to notice that only five benign tumors were included, which may have influenced our results. Several human cancer reports detected significant differences between patients with benign and malignant mammary [47], ovarian [31], and prostatic [32] tumors, which reinforces the hypothesis that the neoplasm histological type can influence uPA concentrations in serum.

Although the difference between groups was not statistically significant, patients with non-metastatic neoplasms had 65% lower uPA values than those with metastasis, suggesting that aggressive neoplasms can secrete higher uPA levels into the circulation. As proposed by several authors [31,32], the proteolytic activity of uPA contributes to the ECM degradation allowing for cancer cells to disseminate and metastasize [6-8]. The lack of statistical significance in this series may be due to its small size and/or to the heterogeneity of the included neoplasms. It may otherwise be explained by biological facts such as low secretion of uPA by the included neoplasms, different secretion of uPA by different histological tumor types, and fast uPA metabolization, and/or elimination from the circulation.

The size of the cutaneous neoplasms was also not related to the values of uPA, unlike previously reported in human [48] and veterinary medicine [35]. Once again, the small sample size may justify this discrepancy.

This study also demonstrated that the presence of low levels of hemolysis in serum samples did not influenced the values of uPA. This information might be useful to avoid the rejection of samples with low hemolysis in future studies.

## Conclusion

This study established for the first time the mean serum concentration of uPA in healthy dogs and cancer patients. These preliminary results do not support the use of uPA concentration in serum as a general screening test to identify dogs with cancer. In addition, uPA serum values were also not useful to differentiate patients with benign and malignant neoplasms. However, there was a tendency for dogs with metastatic neoplasms to present higher uPA serum levels. Future research studies should focus on patients with specific tumor types and/or tumors with similar molecular characteristics, to further determine the prognostic and/or predictive value of serum uPA.

## Authors’ Contributions

SCR conducted the study. AAS, AJdeM, JNR, and SCR conceived and designed the experiment work. SCR, AAS, LRLM, and RRFF collected the samples and clinical data. IV and JNR analyzed the data. AAS and AjdeM revised the manuscript. All authors read and approved the final manuscript.
